# Shedding Lights on the Extracellular Vesicles as Functional Mediator and Therapeutic Decoy for COVID-19

**DOI:** 10.3390/life13030840

**Published:** 2023-03-20

**Authors:** Abhimanyu Thakur

**Affiliations:** Ben May Department for Cancer Research, Pritzker School of Molecular Engineering, University of Chicago, Chicago, IL 60637, USA; abithakur1211@gmail.com

**Keywords:** COVID-19, exosome, extracellular vesicles, theranostic, therapeutics, vaccine

## Abstract

COVID-19 is an infectious disease caused by the novel coronavirus (SARS-CoV-2) that first appeared in late 2019 and has since spread across the world. It is characterized by symptoms such as fever, cough, and shortness of breath and can lead to death in severe cases. To help contain the virus, measures such as social distancing, handwashing, and other public health measures have been implemented. Vaccine and drug candidates, such as those developed by Pfizer/BioNTech, AstraZeneca, Moderna, Novavax, and Johnson & Johnson, have been developed and are being distributed worldwide. Clinical trials for drug treatments such as remdesivir, dexamethasone, and monoclonal antibodies are underway and have shown promising results. Recently, exosomes have gained attention as a possible mediator of the COVID-19 infection. Exosomes, small vesicles with a size of around 30–200 nm, released from cells, contain viral particles and other molecules that can activate the immune system and/or facilitate viral entry into target cells. Apparently, the role of exosomes in eliciting various immune responses and causing tissue injury in COVID-19 pathogenesis has been discussed. In addition, the potential of exosomes as theranostic and therapeutic agents for the treatment of COVID-19 has been elaborated.

## 1. Introduction

The World Health Organization officially named coronavirus infectious disease 2019 (COVID-19) as an infectious disease caused by the severe acute respiratory syndrome coronavirus type 2 (SARS-CoV-2). The occurrence of this disease was first registered at the end of the year 2019, and it has since become a global public health concern [1,2]. In addition to causing a disruption to health care systems, COVID-19 had a significant economic effect worldwide. Clinically, COVID-19 affects the lungs, with most cases exhibiting asymptomatic or mild symptoms; however, in some cases, particularly among the elderly or those with underlying conditions, interstitial pneumonia (IP) or acute respiratory distress syndrome (ARDS) can occur, resulting in a need for mechanical ventilation in intensive care units [3]. Additionally, COVID-19 has been observed to present systemic manifestations, including cardiovascular, gastrointestinal, hematopoietic, renal, and immune system dysfunction, sepsis, multi organ failure, and, in some cases, death [4,5].

SARS-CoV-2 is the seventh human coronavirus discovered to date. It is structurally similar to SARS-CoV and MERS-CoV, both of which lead to acute respiratory diseases [6]. Morphologically, SARS-CoV-2 is a pleomorphic or spherically enveloped virus particle with a diameter range of 80–120 nm, containing a 30 kB positive single-stranded RNA within its membrane and a variety of viral proteins, including its spike (S) protein. Studies have found that the spike protein of SARS-CoV-2 is highly structurally homologous to that of SARS-CoV. In addition, SARS-CoV-2 has been shown to have an increased affinity for the host cell’s angiotensin converting enzyme 2 (ACE2) receptor, which likely contributed to the enhanced infection and transmission potential of the virus [7,8,9]. Alongside SARS-CoV-2, there are four other known human coronaviruses (HCoV-NL63, HCoV-229E, HCoV-OC43, and HKU1), which can cause mild common cold symptoms [10].

Recent reports suggest that the viral infections of the lungs and respiratory tract and the inflammation and injury involve the intracellular communication via extracellular vesicles (EVs). These EVs are composed of different types of vesicles, namely exosomes, microvesicles (MVs), and apoptotic bodies (ABs), based on the size, biogenesis pathway, and markers [11,12]. Exosomes are approximately 30–200 nm in diameter and are released via the endosomal pathway; MVs are generated via budding off from the membrane, and ABs are released from the cells undergoing apoptosis. In general, exosomes and MVs are collectively referred to as EVs [13,14,15]. Furthermore, EVs have the capability to transfer viral particles from infected cells to healthy cells, as well as to modify the body’s immune responses [16]. On the other hand, the plasma of COVID-19 patients was found to contain exosomes enriched with fragments of the SARS-CoV-2 virus, including its spike protein. This suggests that exosomes extracted from the plasma of COVID-19 patients could potentially elicit an immune response to the infection. Studies have shown that these exosomes can stimulate a protective T-cell response and increase B-cell proliferation. Furthermore, these exosomes can be used to generate vaccine-induced antibodies, providing an important tool for the development of effective treatments for COVID-19 [17]. Therefore, it is pertinent to discuss the potential role of EVs as functional mediators and therapeutic decoys for COVID-19.

## 2. How Does SARS-CoV-2 Enter the Host Cells?

**Figure 1:**
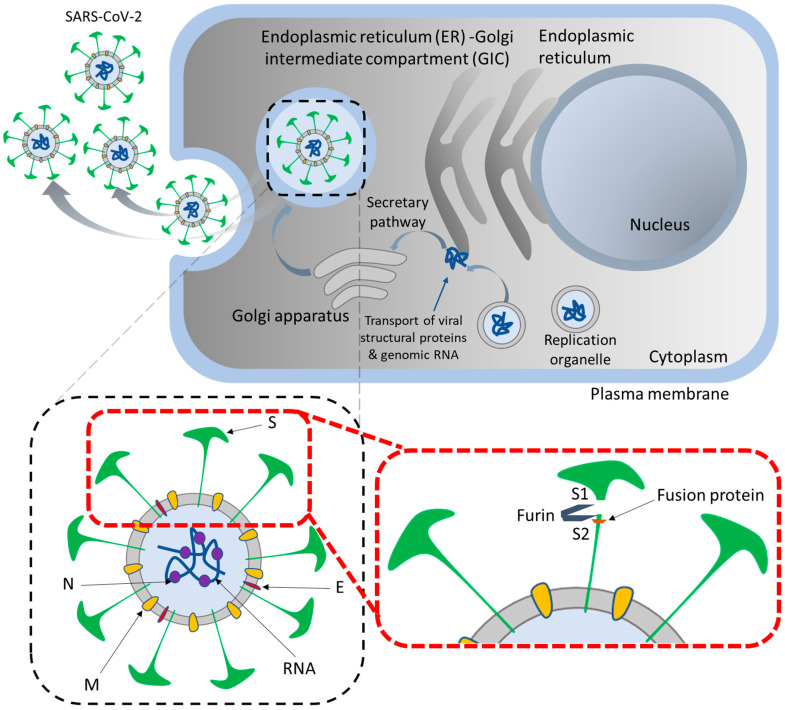
Mechanism of SARS-CoV-2 maturation in the host cells and its structural component. Infection with a coronavirus leads to the formation of ‘replication organelles’ in the perinuclear area, observed by electron microscopy in cells infected with SARS-CoV-2. These are double-membrane structures originating from the endoplasmic reticulum (ER) and containing viral replication complexes. Viruses are composed of four proteins—S (spike), E (envelope), M (membrane), and N (nucleocapsid). The S protein gives the virus the appearance of a ‘crown’. This facilitates key entry steps, including receptor binding and membrane fusion. The S protein is cleaved by furin or furin-like proprotein convertase in the Golgi apparatus into the S1 and S2 subunits. After being synthesized, the viral proteins and RNA are transported to the ER-Golgi intermediate compartment (ERGIC) for assembly and budding. In the target cell, the S1 subunit binds the receptor, and the S2 subunit anchors the S protein to the virion membrane, thus facilitating fusion. The E and M proteins are responsible for assembling the virus and enabling it to bud into the ERGIC lumen, reach the plasma membrane, and be released into the extracellular space.

SARS-CoV-2 is a single-stranded RNA virus that encodes four major protein types: nucleocapsid, membrane, envelope, and spike proteins. The spike protein is a two-part subunit, consisting of S1 and S2, which are crucial for the virus to enter the host cell. S1 binds to the ACE2 receptor, while S2 is cleaved by trans-membrane serine protease-2 (TMPRSS2), permitting the virus to fuse with the cell membrane. The spike is heavily glycosylated, which protects it from the host’s immune system and alters the conformation of the receptor binding domain on S1, thus allowing for binding with ACE2 [18,19]. The virus is most heavily concentrated in tissues that express both ACE2 and TMPRSS2, such as the tracheobronchial tree, alveolar type II (AT2) cells, endothelial cells, cardiomyocytes, and the small bowel and colon epithelial cells [20]. This distribution of cell susceptibility partially explains the array of symptoms seen in COVID-19 patients. The expression of ACE2 and Cathepsin L in alveolar type 1 pneumocytes (AT1 cells) and AT2 cells, enterocytes, cardiomyocytes, and the placenta is linked to the activation of IL-1β and NLRP3-mediated inflammatory cascades leading to cell apoptosis or pyroptosis, as well as the remodeling of extracellular matrices and the recruitment of neutrophils for emergency myelopoiesis [21,22]. Heparan sulfate aids in the attachment of viral particles to the cell surface, while the presence of cleavage sites on the spike protein, which are similar to furin, promote viral replication in the lung [23,24]. Neuropilin1 (NRP1) binds these furin-derived substrates, aiding in the entry of the virus into nasal cells [25,26]. Figure 1 illustrates the mechanism of SARS-CoV-2 maturation into the host cells and its major components.

## 3. Implications of SARS-CoV-2 Infection

SARS-CoV-2 infection induces pathologic changes in the immune system, leading to tissue injury and presenting as three distinct groups of clinical manifestations. These include humoral immunodeficiency with B-cell defects, hyperinflammation with T-cell subset loss and elevated cytokines (IL-6, IL-1β, TNF-α), and complement-driven injury associated with neutrophil extracellular traps (NETs) and systemic thrombosis [27,28].

### 3.1. Interferon-Associated Reactions

Interferon (IFN) plays an important role in the body’s defense against viral infections. It is divided into three categories: type I binds to the IFN-α/β receptor, type II (IFN-γ) is activated by IL-12, and type III is signaled through either IL-10R2 or IFNLR-1 complexes. In healthy people, these responses are beneficial; however, if the IFN response is too strong or too late, it can cause tissue damage. Type I IFN levels in peripheral blood mononuclear cells usually increase soon after a viral infection and then decrease as the infection is cleared. People with severe COVID-19, however, may display reduced or delayed IFN responses, which could be due to genetic factors, antibodies that neutralize the IFN, or a decrease in the number of plasmacytoid dendritic cells that produce TLR7, a toll-like receptor that recognizes single-stranded viral ribonucleic acids and initiates IFN responses [29,30].

The levels of IFN in different tissues of COVID-19 patients vary and are associated with the severity of their disease. Type I IFN plays an important role in the early stages of infection and can be seen in those with sustained viral replication. In patients with severe disease, increased levels of type II IFN were found in all sites, whereas type III IFN was mostly present in the upper airways of those with mild disease and high viral loads. Animal models suggest that the failed IFN-driven antiviral response may be the cause of serious COVID-19. In ferrets with SARS-CoV-2 pneumonia, the virus is usually cleared as a result of an early M1 macrophage response (IL-1β, CXCL8 and type I IFN) followed by an anti-inflammatory M2 macrophage response. However, aged ferrets with higher viral loads in the respiratory tract have a more serious disease and an M1 macrophage response that does not lead to the M2 macrophage-mediated resolution phase. This continuous CXCL10 secreted by M1 macrophages attracts monocytes/macrophages, NK cells, and T cells, thus promoting an inflammatory state [21,31].

### 3.2. Cytokine Release

SARS-CoV-2 infection has been shown to cause increased levels of neutrophil degranulation and cytokine expression compared to influenza. Examples of cytokines that can be found at elevated levels include IL-1β, IL-1RA, TNF-α, G-CSF, CCL7, CXCL1, CXCL8, CXCL11, and CXCL12a. In some cases, macrophage activation syndrome can be triggered, characterized by an increase in type II interferon-related responses, as well as elevated levels of IL-6, IL-1β, TNF-α, and ferritin. Severe cases of COVID-19 are associated with prolonged, heightened levels of cytokines, such as IL-6, IL-8/CXCL8, CXCL9, CXCL10, TNF-α, MCP1/CCL2, RANTES/CCL5, IL-18, and MIP-1α/CCL3, which can last for up to two months [21,32,33].

### 3.3. Effect of COVID-19 on Innate and Adaptive Immunity

Evidence suggests that dysregulation of the immune response in COVID-19 patients can lead to viral hyperinflammation, which increases their mortality. Evaluation of hyperinflammation using laboratory parameters may help to reduce mortality in these patients [34,35].

Innate immune cells sense antigenic components known as pathogen-associated molecular patterns (PAMPs) through receptors such as C-type lectin receptors, NOD-like receptors (NLRs), RIG-I-like receptors (RLRs), and Toll-like receptors (TLRs) to detect pathogens. Coronaviruses, which are a kind of RNA virus, are identified through cytoplasmic and endosomal RNA sensors, including RIG-I and TLRs (TLR2, TLR3, and TLR7) [36]. Polyinosinic-polycytidylic acid (poly I:C) has been shown to prevent coronavirus infection when activated with TLR3. The recognition of RNA viruses by TLRs and RIG-1 activates transcription factors such as nuclear factor kappa-light-chain-enhancer of activated B cells (NF-kB) and interferon regulatory factor (IRF) 3, which then travel to the nucleus and cause the expression of pro-inflammatory cytokines, chemokines, and type I IFN. Type I interferons (IFNs) are the first defense against viral intrusions. Upon engaging IFNAR (IFNα/β receptor) signaling, JAK1 and TYK2 phosphorylate STAT1 and STAT2 molecules, forming a complex with IRF9. This complex then moves to the nucleus, prompting the transcription of IFN-stimulated genes (ISGs). These ISGs consequently express antiviral proteins such as IFITM1, 2, and 3, which are effective against SARS-CoV [37,38,39,40,41,42,43,44,45].

The adaptive immune system plays a crucial role in controlling SARS-CoV replication and disease severity. T cells, which are divided into CD4+ and CD8+ T cells, are essential for this purpose, as they stimulate the production of pathogen-specific antibodies and eliminate virus-infected cells. Memory CD8+ T cells have been shown to be particularly important in protecting hosts against SARS-CoV, as they produce cytokines and cytolytic molecules. In contrast, a T-cell depletion or exhaustion is linked to more severe symptoms and mortality in those suffering from COVID-19 and is associated with an increased production of Th17, Th1, and GM-CSF cytokines. The role of humoral immunity in regulating CoV infections is not yet fully understood. Research has shown that SARS-CoV triggers antibody production in the form of both IgM and IgG. IgG antibodies are known to be more enduring than IgM, suggesting a potential role in the provision of protective immunity [46,47,48,49].

### 3.4. Tissue Injury in COVID-19 via Neutrophil Extracellular Traps and Thrombosis

NETs are three-dimensional lattice structures made up of decondensed chromatin strands, containing citrullinated histones, antimicrobial proteins, and cytokines. When neutrophils undergo apoptosis, they release NETs which are able to capture and destroy pathogens, as well as induce the production of pro-inflammatory cytokines such as IL-6, IL-8/CXCL8, RANTES/CCL5, and platelet factor 4. SARS-CoV-2 has been found to directly stimulate NET formation, which can become misregulated and lead to cell damage. Additionally, NETs can create a positive feedback loop that can result in the aggregation of platelets, the release of cytokines, and the activation of complement. COVID-19 patients have been observed to have higher levels of NETs in their plasma, tracheal aspirates, lung tissue, and arterial thrombi when compared to those with other pulmonary diseases. Histological findings of NETs with micro-thrombosis have been linked to severe cases of COVID-19 pneumonia. Consequently, targeting NET formation may become a potential treatment option for critically ill COVID-19 patients [50,51,52,53,54].

People with COVID-19 and ARDS are at risk of systemic damage to the endothelium and thrombosis in both the arterial and the venous systems. Compared to those with influenza, patients with COVID-19 are more likely to experience endothelial injury and thrombosis in the lungs. Markers of systemic injury, such as CRP, ESR, fibrinogen, and pro-calcitonin, are usually increased in these patients, and they also have higher rates of bleeding. The implementation of anticoagulation as a part of COVID-19 treatment has been shown to reduce the one-month mortality rate while avoiding an increased risk of bleeding [55,56,57].

## 4. Role of EVs in COVID-19 Infection and Associated Pathogenesis

**Figure 2:**
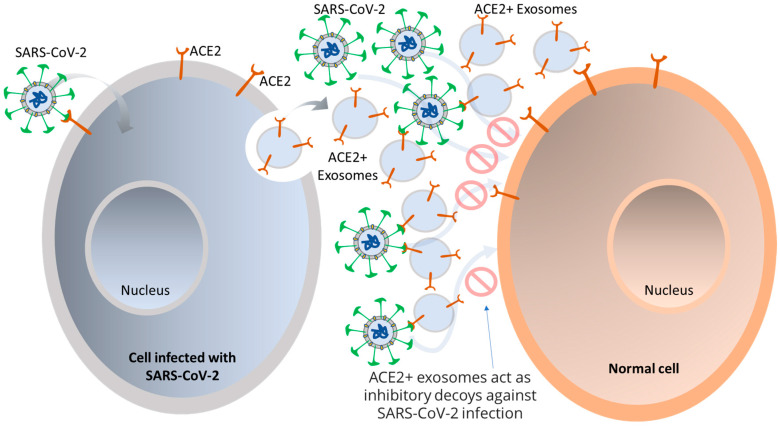
Utilizing EVs from SARS-CoV-2 as a potential therapeutic for COVID-19. Upon SARS-CoV-2 infection, the generation of exosomes with ACE2 on their surface is dependent on TLR signaling and ATG16L1. These ACE2-positive exosomes may act as an effective defense mechanism by binding SARS-CoV-2 and inhibiting its interaction with host cells. In critically ill COVID-19 patients, there is considerable disparity between individuals in terms of the abundance of ACE2-positive exosomes. Increased abundance of these exosomes was associated with shorter hospital stays and fewer days of mechanical ventilation, implying that ACE2-positive exosomes may have a protective role against SARS-CoV-2 in humans.

Viruses access host cells via cell-surface receptors and can even transfer these receptors to receptor-null cells, making them more infectious [58,59]. Integrins, in particular, are suitable for attaching and/or entering both enveloped and non-enveloped viruses. For example, SARS-CoV has a binding motif of Arg-Gly-Asp (RGD) that it uses to bind to integrins, allowing for an alternate route of viral transmission [60]. Exosomes, which are known to have various adhesion molecules, are believed to bind to target cell membranes and enter via tetraspanin-enriched microdomains (TEMs). Studies on CoV proteolytic priming suggest that blocking the tetraspanin function could be a potential way to prevent infection. SARS-CoV-2 is believed to bind to respiratory epithelial cells and replicate in the airways and alveolar epithelial cells, inciting an immune response. Infected cells are thought to produce exosomes containing viral antigens, self-antigens, and 20S proteasomes [16,61,62]. Exosomes can carry the ACE2 receptor to recipient cells, enabling viral entry, and viruses can use them as a pathway for intra-host spreading. It has been observed that human lung epithelial cells, which are susceptible to SARS-CoV-2 infection, can release exosomes with viral components that can facilitate the transmission of SARS-CoV-2 RNA into human induced pluripotent stem cell-derived cardiomyocytes (hiPSC-CMs), resulting in increased expression of inflammation-related genes in the hiPSC-CMs. Furthermore, exosomes carrying the ACE2 receptor from both healthy donors and recovered COVID-19 patients have been found to reduce SARS-CoV-2 infection by blocking the binding of the viral S protein to its receptor [63]. This phenomenon suggests that ACE2+ exosomes can act as an inhibitory decoy, which could potentially be used as a therapeutic for treating COVID-19, as illustrated in Figure 2. This is akin to the manner in which HIV packages its proteins and RNA into vesicles to spread to non-infected cells, and further investigation is necessary in the context of SARS-CoV-2 [64,65].

During viral infection, EVs are internalized by recipient cells and play an important role in activating the innate immune system, modulating host defense, and evading immune detection [66,67]. The EVs emitted by infected cells are distinct from those of healthy cells and include biomolecules such as RNA, lipids, and proteins. SARS-CoV-2 is one such virus which spreads through exosomes and has been found in vacuoles inside host cells. Its assembly is similar to that of SARS-CoV and close to the rough endoplasmic reticulum [66,67,68]. Bioinformatics analysis has uncovered the presence of proteins involved in coagulation, transport, complement, protease inhibitor, and defense/immunity activities in exosomes. Research on exosomes derived from cells infected with respiratory syncytial virus has discovered that these exosomes activate an innate immune response, stimulating the release of cytokines and chemokines from monocytes and airway epithelial cells [69,70]. Patients suffering from coronavirus infections, such as SARS and MERS, experience high levels of pro-inflammatory cytokines and chemokines, leading to pulmonary inflammation. Severe COVID-19 and mortality have been linked to platelet degranulation, low platelet count, and increased levels of IL-6. The presence of cytokines such as TNF, IL-1β, and IL-6 in SARS-CoV-2 infection may be associated with exosomes, potentially contributing to a cytokine storm and tissue damage [71].

## 5. EVs as Tool for Diagnosis of COVID-19

Exosomes and extracellular RNAs (exRNAs) are involved in numerous pathological processes. ExRNAs, containing molecules such as mRNAs, miRNAs, small nuclear RNAs, transfer RNAs, and long non-coding RNAs (lncRNAs), are released during anti-viral responses and play a role in regulating the innate immune system of the host organism [72]. For COVID-19, early biomarkers, such as those linked to EV proteins, blood coagulation related markers, and liver damage, have been identified, with EV coatomer protein complex subunit beta 2 (COPB2) having the greatest predictive value for severe disease [73,74]. Exosomes are advantageous biomarkers for detecting infection from limited sample sources as they can be easily isolated and stored. Exosomal miRNA is used to monitor chronic infections of HBV and HCV, the latter of which alters the miRNA cargo of exosomes [75].

Culturing SARS-CoV-2 sub-genomic RNAs in Vero cells revealed high levels of specific proteins, including S, Orf3a, E, M, Orf6, Orf7a, and N, and low levels of Orf7b, which can be used as biomarkers for the disease [76]. Additionally, Wölfel et al. found that the presence of the E gene sub-genomic RNA indicates active viral infection and transcription [77]. On the other hand, Alexandersen et al. detected SARS-CoV-2 sub-genomic RNAs in diagnostic samples, which may not necessarily point to active virus replication or infection [78]. Moreover, SARS-CoV-2-derived EVs have been shown to increase the levels of circulating tissue factor (TF)-positive EVs, thus potentially contributing to thrombosis in patients with COVID-19 [73]. Additionally, electron microscopy studies have uncovered the early formation and accumulation of EVs containing viral replication complexes associated with SARS-CoV-2 [79]. Exosomes isolated from patient samples contain various proteins which may be used as potential biomarkers, such as fibrinogen, fibronectin, complement C1r subcomponent, and serum amyloid P-component. It has also been suggested that circulating exosomes may be involved in inflammatory, coagulant, and immunomodulatory processes in COVID-19 [80].

## 6. Therapeutic Applications of EVs for COVID-19

Exosomes, while believed to be involved in the transmission of SARS-CoV-2, may conversely be an advantage for the treatment of COVID-19. To curb the spread of the virus, the uptake of exosomes by neighboring cells could be impeded. Consequently, various treatments for COVID-19 have been proposed, including mesenchymal stem cells (MSCs) and their resulting exosomes. MSCs can generate a variety of cytokines and paracrine factors which can interact with immune cells such as T cells, B cells, dendritic cells, macrophages, and natural killer cells, which could help to reduce the cytokine storm associated with COVID-19. Clinical research has indicated that human umbilical cord-derived MSCs (HUMSCs) could be of benefit in improving the pulmonary function of those with SARS-CoV-2-related pneumonia [81,82]. An alternative approach to MSCs to treat COVID-19 is through secretome-based therapy and exosomes isolated from cell secretome, with exosomes being deemed more effective [83,84,85]. Studies have revealed that exosomes derived from MSCs are non-toxic and can have a similar effect to their parental cells in models of acute and chronic lung injury, sepsis, and ARDS, suggesting that they could be more successful than MSCs in constraining the inflammatory response of COVID-19 [86,87].

Investigations have demonstrated that utilizing exosomes in a clinical environment can be advantageous, with some research indicating that exosomes can be more effective than parent cells in diminishing lung injury in ARDS [88]. Moreover, exosomes can reduce the cytokine storm related to ARDS and increment the anti-inflammatory signaling mediators, thereby lessening the seriousness of lung injury. The use of exosomes has additionally been analyzed for treating COVID-19, where convalescent plasma containing exosomes is used as an immunomodulator [89]. Numerous restorative techniques have been created to treat COVID-19, such as antivirals, anti-infection agents, biologics, immunizations, and convalescent plasma. The adequacy of these medicines is constrained by the high rate of mutation of SARS-CoV-2, which impedes the body’s normal immune response. Plasma treatment has been exhibited to be powerful in seriously sick patients and focusing on the SARS-CoV-2 S protein utilizing neutralizing antibodies is another conceivable treatment. Cytokine treatment, interferon-α2b, and small interfering RNAs have likewise been utilized to repress viral replication [90].

Exosomes are of great interest for therapeutic applications due to their high bioavailability, biocompatibility, and low immunogenicity. This has accelerated research into the use of EVs for the treatment of COVID-19, with a particular focus on early detection and novel therapies. Additionally, exosomes are composed of double lipid bilayers and are capable of transporting various biomolecules which are involved in both physiological and pathological processes, including those of the host immune response. Several studies have highlighted the importance of exRNAs as biomarkers and potential therapeutic targets for COVID-19, emphasizing the necessity of identifying exRNAs for the development of effective treatments [87].

Exosomes, small vesicles containing microRNA, proteins, and cytokines, have been proposed as a promising therapeutic approach to reduce inflammation and improve the outcomes of severe COVID-19 patients [89]. Bone marrow-derived exosomes have been demonstrated to reduce alveolar inflammation and support edema clearance, as well as restore the permeability of epithelial membranes in various animal models of acute lung injury, ARDS, asthma, and other inflammatory diseases [91]. Additionally, miRNA-155 mimic-loaded exosomes were found to increase miRNA-155 levels in primary mouse hepatocytes and the liver of miRNA-155 knockout mice, while significantly reducing and preventing the production of TNFα and the expression of SOCS1 mRNA in RAW macrophages, respectively [92]. MSC-derived exosomes have been demonstrated to protect lung epithelial cells against oxidative stress-induced cell death [93,94]. Animal studies have also shown that MSC-derived exosomes can increase the proliferation of lung epithelial cells and modulate the phenotype and function of lung-infiltrating dendritic cells. Systemic administration of MSC-derived exosomes has been found to decrease Escherichia coli endotoxin-induced acute lung injury in a mouse model and to guard the brain against sepsis-induced injury in rats [95,96]. Moreover, these exosomes have been observed to reduce endothelial cell apoptosis, attenuate the cytokine storm caused by SARS-CoV-2, and decrease IL-6 production, while increasing IL-10 production [88,97].

## 7. Targeting Exosomal Pathways Pharmacologically as COVID-19 Therapy

Pharmacological inhibitors of exosomes are a potentially effective treatment to combat viral infections such as SARS-CoV-2. Examples of these inhibitors include GW4869, Dynasore, calpeptin, Manumycin A, Y27632, Imipramine, and pantethine [98]. The primary way these inhibitors work is by blocking the release of viral extracellular vesicles and microvesicles, as well as by preventing the EVs’ movement or lipid metabolism. Experiments have revealed these inhibitors to be capable of decreasing the production of exosomes and microvesicles and inhibiting SARS-CoV-2 replication in vitro. As a result, they could have therapeutic potential in the case of COVID-19 [58].

## 8. Utilizing EVs as a Delivery Tool for COVID-19 Therapy

EVs have been demonstrated to be a reliable delivery vector for drugs due to their ability to reach target tissues, cross biological barriers, and protect their cargo from the immune system and degradation. Compared to synthetic delivery systems, exosomes have several advantages, including a more natural presence in bodily fluids, enhanced stability under physiological conditions, and less toxicity and immunogenicity [15]. Exosomes are also able to deliver specific cargo to recipient cells and cross the blood–brain barrier to deliver materials to the brain [99,100]. miRNAs and various drugs have been encapsulated into exosomes and used to target molecules within infected cells to reduce local inflammation or prevent apoptosis. One study found that pre-treating mesenchymal stem cells with IL-1β augmented their immunomodulatory effects, enabling their exosomes to transfer miR-146a to target cells.

Exosomes derived from MSCs, being equipped with immunomodulatory components, have been proposed as a potential treatment option for COVID-19 in combination with antiviral drugs [101,102]. It has been found that MSC-derived exosomes delivered via inhalation had beneficial effects on lung repair in patients affected by the virus [103]. Moreover, Gunasekaran et al. highlighted the role of exosomes released from infected cells in the infection and spread of SARS-CoV-2 [104]. Considering their drug delivery capabilities and anti-inflammatory effect and their capacity to regenerate damaged tissues, exosomes may be used as therapeutics for COVID-19, although further research and clinical trials are needed to assess their efficacy and safety [105,106].

## 9. Utilizing EVs for Developing COVID-19 Vaccine

Exosomes secreted by cells also have the potential to be used as a virus-free vaccine against COVID-19. These exosomes can be engineered to contain antigens, resulting in a strong CD8+ T-cell and B-cell response, and they have been found to have a low basal immunogenic profile and to be safe according to in vitro studies and animal models [107]. Furthermore, exosomes have been demonstrated to trigger antigen-specific CD8+ T-cell responses, such as the release of interferon gamma (IFN-γ). This IFN-γ production is an indication of an active cellular immune response, which further supports the development of exosomes as a vaccine against COVID-19 [108]. Research has shown that exosome-based vaccines induce higher levels of antibodies than serum samples taken from SARS patients, suggesting that exosome-based vaccines may be beneficial in the treatment of COVID-19 [69,109]. Additionally, the insertion of SARS S protein into exosomes has created a chimeric protein, which has been found to increase neutralizing antibody titers when combined with a SARS coronavirus spike vaccine and an adenoviral vector vaccine. However, exosomes from virus-infected cells can also promote viral infection and inhibit immune cell responses [88].

Currently, vaccines are developed utilizing inactivated or debilitated forms of viruses, emphasizing the viral S protein, as well as alternative techniques, including vaccines based on viral vectors, mRNA, or the full-length or partial S protein [110,111]. Two mRNA-based vaccines, BNT162b2 (developed by Pfizer and BioNTech) and mRNA-1273 (developed by Moderna), have been approved by medical regulatory authorities in the UK, USA, and EU, resulting in a mass vaccination campaign being initiated [112]. In addition, the UK has also approved ChAdOx1 nCoV-19, a vaccine developed by AstraZeneca and Oxford University [113]. To further the development of COVID-19 vaccines, biotechnology companies have been creating exosome-based vaccines, such as that of Capricor Therapeutics, containing mRNA for the full-length S protein and modified S, N, M, and E proteins, Allele Biotechnology and Pharmaceuticals’ mRNA-based vaccine, and the Ciloa’s CoVEVax vaccine, which consists of EVs containing the full S protein. Codiak BioSciences and Versatope Therapeutics have also been developing vaccines using engineered EVs [114].

## 10. Conclusions and Future Outlooks

The COVID-19 pandemic has affected millions of people worldwide; it was caused by the SARS-CoV-2, a single-stranded positive-sense RNA virus. The primary mode of entry for the virus is through the ACE-2 receptors present on the surface of the host cells. Recently, other receptor proteins, including CD147, NRP1, and TMPRSS2, have been reported to mediate the host cell entry of SARS-CoV-2. Once the virus binds to the receptor, it can use the endocytic pathway to enter the cell and initiate its replication. Apart from the direct entry, the virus can also enter the host cells via EVs. EVs are nano-sized membranous vesicles that are released by cells and can carry proteins, lipids, and nucleic acids. Infection with SARS-CoV-2 can lead to a variety of implications, including interferon-associated reactions, cytokine release, and tissue injury due to neutrophil extracellular traps and thrombosis. EVs have been found to play an important role in the infection and associated pathogenesis of COVID-19. EVs can be used as a tool for diagnosis, as well as for therapeutic applications. Additionally, EVs can be used as a delivery tool for targeted pharmacological therapies, as well as for developing COVID-19 vaccines. The role of EVs in COVID-19 has been extensively studied, and recent research has shed light on the functional mediator and therapeutic decoy capabilities of EVs. EVs can be used to deliver therapeutic molecules, such as antibodies, to the infected cells, thus serving as a protective shield against the virus. Furthermore, EVs can be used for developing a COVID-19 vaccine, which has the potential to be a successful and efficient approach for treating and preventing the disease. Apparently, in addition to the role of EVs as functional mediators in COVID-19 infection and pathogenesis, EVs have been identified as a potential therapeutic option for treating and preventing COVID-19. Further research is needed to better understand the role of EVs in the infection and associated pathogenesis of COVID-19 and to determine the most effective ways to use EVs for treating and preventing the disease.

COVID-19 has raised many questions and directions for further research. One of the most pressing questions is how to prevent the spread of the virus. Are there any treatments that could improve the outcome of the infection? Is there a way to develop a vaccine to prevent the spread of the virus? Another area of research is the long-term effects of the virus. What will be the impact on the health of individuals who have been infected? Will there be any long-term effects on the immune system? Are there any potential chronic conditions that could arise from the infection? A third research direction is the economic impact of the virus. How has the virus affected the economy? What can be done to mitigate the economic damage caused by the virus? Finally, there is the question of how to best manage the public health response to the virus. What strategies should be used to contain its spread? How should governments and health organizations collaborate to make public policy considering the scientific evidence to address the challenges posed by the virus? These are just some of the emerging questions and research directions related to COVID-19. There is a great deal of work to be done to understand the virus and its potential impacts. It is important that researchers continue to explore these questions to effectively manage the virus and protect the public.

## Data Availability

Not applicable.
